# The role of inflammatory biomarkers RBP4, Lipocalin-2, and hsCRP in prediabetes and type 2 diabetes mellitus

**DOI:** 10.5937/jomb0-57001

**Published:** 2025-08-21

**Authors:** Diker Vesile Ornek, Ozden Serin, Ertug Ebru Yorulmaz, Ozlem Baytekin, Osman Oguz, Güvenç Güvenen

**Affiliations:** 1 Mehmet Akif Ersoy T.C.S. Training and Research Hospital, Medical Biochemistry, Istanbul, Turkey; 2 Istanbul Taksim Egitim ve Aragtirma Hastanesi, Medical Biochemistry, Istanbul, Turkey; 3 Istanbul Kartal Egitim ve Aragtirma Hastanesi, Medical Biochemistry, Istanbul, Turkey; 4 §igli Hamidiye Etfal Egitim ve Aragtirma Hastanesi, Medical Biochemistry, Istanbul, Turkey; 5 American Hospital, Clinical Biochemistry, Istanbul, Turkey; 6 Bezmiâlem Vakif Üniversitesi, Medical Biochemistry, Istanbul, Turkey

**Keywords:** type 2 diabetes mellitus (T2DM), impaired fasting glucose (IFG), impaired glucose tolerance (IGT), retinol-binding protein 4 (RBP4), lipocalin-2 (LCN2), high sensitivity c-reactive protein (hsCRP), inflammation, insulin resistance, adipocytokines, dijabetes tipa 2 (T2DM), poremećena glikemija natašte (IFG), poremećena tolerancija na glukozu (IGT), protein vezan za retinol 4 (RBP4), lipokalin-2 (LCN2), visokosenzitivni C-reaktivni protein (hsCRP), upala, insulinska rezistencija, adipocitokini

## Abstract

**Background:**

Adipocytokines, along with macrophages infiltrating adipose tissue, contribute to chronic low-grade inflammation, which leads to insulin resistance and type 2 diabetes. Understanding insulin resistance in non-diabetic individuals and its cellular mechanisms is key for developing effective treatments and improving current protocols. This study aimed to investigate the levels of Retinol Binding Protein 4 (RBP4), Lipocalin-2, and high-sensitivity C-reactive protein (hsCRP) in individuals with impaired fasting glucose (IFG), impaired glucose tolerance (IGT), and type 2 diabetes mellitus (T2DM).

**Methods:**

This prospective case-control study included individuals with IFG, IGT, and newly diagnosed T2DM. Routine laboratory tests, including fasting blood glucose, insulin, HbA1c, and lipid profiles, were collected and analysed. RBP4, Lipocalin-2, and hsCRP levels were measured using the ELISA method.

**Results:**

Significant differences were found in fasting blood glucose, insulin, triglycerides, total cholesterol, and HOMA-IR values among the study groups. hsCRP levels were significantly elevated in the IGT and T2DM groups compared to controls, while RBP4 and Lipocalin-2 levels showed no significant differences. A positive correlation was observed between hsCRP and HbA1c in the IFG group, as well as between hsCRP and Lipocalin-2 in the T2DM group.

**Conclusions:**

This study demonstrates that hsCRP and Lipocalin-2 are associated with early glucose metabolism abnormalities and may serve as markers for insulin resistance and inflammation in prediabetes and T2DM. Future research is needed to clarify the roles of these biomarkers and their potential as therapeutic targets in diabetes prevention and treatment.

## Abstract (sr)

**Uvod:**

Adipocitokini, zajedno sa makrofazima koji infiltriraju masno tkivo, doprinose hroničnoj upali niskog stepena, što dovodi do insulinske rezistencije i dijabetesa tipa 2. Razumevanje insulinske rezistencije kod osoba bez dijabetesa i njenih ćelijskih mehanizama je ključno za razvoj efikasnih terapija i poboljšanje postojećih protokola. Ova studija je imala za cilj da ispita nivoe proteina vezanog za retinol 4 (RBP4), lipokalina-2 i visokosenzitivnog C-reaktivnog proteina (hsCRP) kod osoba sa poremećenom glikemijom nataste (IFG), poremećenom tolerancijom na glukozu (IGT) i dijabetesom tipa 2 (T2DM).

**Metode:**

Ova prospektivna studija slučaja i kontrole je uključila ispitanike sa IFG, IGT i novodijagnostifikovanim T2DM. Prikupljeni su i analizirani rutinski laboratorijski parametri, uključujući glikemiju nataste, insulin, HbA1c i lipidni profil. Nivoi RBP4, lipokalina-2 i hsCRP određeni su ELISA metodom.

**Rezultati:**

Utvrđene su značajne razlike u nivoima glukoze nataste, insulina, triglicerida, ukupnog holesterola i HOMA-IR među ispitivanim grupama. Nivoi hsCRP-a su bili značajno poviseni kod ispitanika sa IGT i T2DM u poređenju sa kontrolnom grupom, dok RBP4 i lipokalin-2 nisu pokazali značajne razlike. Uočena je pozitivna korelacija između hsCRP-a i HbA1c u IFG grupi, kao i između hsCRP-a i lipokalina-2 u T2DM grupi.

**Zaključak:**

Ova studija pokazuje da su hsCRP i lipokalin-2 povezani sa ranim poremećajima metabolizma glukoze i mogu da posluže kao markeri insulinske rezistencije i upale kod predijabetesa i T2DM. Potrebna su buduća istraživanja kako bi se razjasnila uloga ovih biomarkera i njihov potencijal kao terapijskih ciljeva u prevenciji i lečenju dijabetesa.

## Introduction

Diabetes Mellitus (DM) is a metabolic disease that causes disruptions in carbohydrate, protein and fat metabolism due to insulin deficiency or inefficiency. Type 2 DM (T2DM) constitutes more than 90% of all diabetes cases. According to International Diabetes Federation (IDF) data, 10.5% of the adult population between the ages of 20-79 years have DM [1]. While 5 in 4 adults with DM live in low- and middle-income countries. On the other hand, impaired glucose tolerance (IGT) and impaired fasting glucose (IFG), which are transition states between normal glycaemia and DM, are significant risk factors for the progression to DM, whereas IGT is also an independent risk factor for cardiovascular complications [2].

The global rise in obesity and related diseases has increased the interest in adipose tissue, which functions as both a metabolic and endocrine organ. Current research indicates that adipose tissue is not only a lipid storage site but also key for energy homeostasis, endocrine regulation, metabolic functions, and inflammatory processes [3] [4]. Adipocytes secrete various bioactive proteins known as adipocytokines, which are vital for maintaining energy balance and are potential targets for obesity treatment and associated conditions. Adipocytokines from adipocytes and macrophages infiltrating adipose tissue contribute to a low-grade chronic inflammatory state, leading to insulin resistance and type 2 DM [5]. Identifying insulin resistance in non-diabetic individuals and understanding its cellular mechanisms will aid in developing effective treatments and optimising existing protocols.

Inflammation is an organism's response to exogenous foreign substances or endogenous pathological disorders. Inflammation is known to increase significantly in T2DM and plays a vital role in the development of vascular complications of diabetes [6]. However, it is not yet understood whether these mechanisms are a cause or a result of the natural course of T2DM.

Retinol-binding protein 4 (RBP4) is a member of the lipocalin family and the main transport protein for retinol (vitamin A) in the bloodstream, with the highest expression in the liver. It plays a crucial role in retinoid homeostasis by binding to retinol for its mobilisation from the liver. Beyond its role in vitamin A transport, RBP4 has been linked to insulin resistance since 2005, when research showed increased RBP4 levels in insulin-resistant mouse models and decreased susceptibility to insulin resistance in RBP4-deficient mice [7] [8]. Overexpression or injection of RBP4 caused glucose intolerance and insulin resistance, suggesting RBP4 acts as an adipokine connecting obesity with insulin resistance [9]. Elevated RBP4 levels in humans correlate with increased adipose RBP4 mRNA, intra-abdominal fat, and decreased insulin sensitivity. In obesity and T2DM, RBP4 is elevated in adipose tissue, contributing to systemic insulin resistance and inflammation through Toll-like receptor-4 (TLR4) [10]. This »sterile inflammation« indicates that RBP4 may play a causative role in endothelial and vascular inflammation, potentially contributing to cardiovascular disease and diabetic microvascular complications.

Lipocalin-2 (LCN2), also known as neutrophil gelatinase-associated lipocalin (NGAL), siderocalin, and 24p3, is a multifunctional protein that is involved in various physiological and pathological processes. It has been reported to deliver iron to cells, leading to intracellular iron overload, oxidative stress, cellular degeneration, and increased levels of advanced glycation end-product (AGE) receptors. LCN2 also activates metalloproteinase-9 (MMP-9) by forming a stable complex with it [11]. Recent clinical studies have shown a strong link between LCN2 expression and the risk of impaired glucose metabolism. Additionally, elevated LCN2 levels have been associated with diabetic complications such as retinopathy and nephropathy [12] [13].

C-reactive protein (CRP) is produced in the liver in response to an acute inflammatory stimulus and is linked to metabolic syndrome and coronary artery disease. CRP inhibits endothelial nitric oxide production and promotes monocyte recruitment into atheromatous plaques, causing plaque instability [14]. High sensitivity CRP (hsCRP) is a sensitive form of CRP detectable between 0.01 mg/Lto 10 mg/L. Evidence suggests that hsCRP levels above 2 mg/L indicate residual inflammatory risk [15]. Elevated hsCRP is associated with increased cardiovascular events in T2DM patients and the development of disease in non-diabetic individuals, indicating a higher risk across all levels of metabolic syndrome [16].

Hence, this study aimed to measure RBP4, LCN2 and hsCRP levels in individuals with IFG, IGT, and newly diagnosed T2DM and to examine their relationship with insulin resistance and inflammation.

## Materials and methods

This was a prospective case-control study conducted in one of the largest training and research hospitals in a European metropolitan city. This study was approved by the Ethics Committee of Istanbul Training and Research Hospital Clinical Research (Date: 2009/12/25, No: 25005), and informed consent was obtained from all participants. The study was conducted in accordance with the Declaration of Helsinki.

The internal medicine physicians from the hospital's outpatient clinics recruited patients. Among those who applied to the physician, individuals with fasting blood glucose (FBG) levels between 100-125 mg/dL who had not been diagnosed with diabetes and had not started medical treatment were directed to the Clinical Biochemistry Laboratory for oral glucose tolerance testing (OGTT) according to the 2005 ADA criteria [17].

OGTT was performed with 75 g of glucose solution dissolved in 500 mL of water after 10-12 hours of overnight fasting. At the end of the 2-hour waiting period, a blood sample was obtained.

IGT was defined as having normal fasting plasma glucose and plasma glucose levels of 140-199 mg/dL following the OGTT procedure. IFG was defined as having fasting plasma glucose of 100-125 mg/dL and normal 2-hr post-OGTT plasma glucose levels.

Exclusion criteria included conditions affecting the endocrine system, kidney and liver disease, acute infections, and medications that may potentially affect insulin secretion and sensitivity. Among the patients who were willing to participate, those under the age of 51 and over the age of 65 were also excluded.

Patients were stratified into three groups - IFG, IGT, and T2DM - according to the 2005 ADA criteria, based on FBG and 2-hour plasma glucose levels post-OGTT. A control cohort comprised 20 individuals exhibiting FBG levels below 100 mg/dL without other clinical conditions.

Patient demographics, body mass index (BMI), and waist-to-hip ratio of participants were collected and recorded.

Venous blood samples were collected into clot-activated and K-EDTA anticoagulant-containing tubes and centrifugated within one hour following the collection. Routine biochemistry tests, including FBG, insulin, HbA1c, and lipid profile, were analysed on the same day of withdrawal. Samples for the study variables were stored at -80°C for 4-6 weeks until the day of analysis.

Serum RBP4 (AssayPro, MO, USA), LCN2 (Quantikine, MN, USA), and hsCRP (CALBIOTECH, El Cajon, CA, USA) levels were determined by the ELISA (Enzyme-Linked Immunosorbent Assay) method based on the sandwich model.

RBP4 and LCN2 levels were expressed in ng/mL, whereas hsCRP levels were expressed in mg/L.

### Statistical analysis

UNISTAT version 5.1 (Unistat Ltd, Highgate London, UK) was used for statistical analysis. The normality of the data was assessed using the Kolmogorov-Smirnovtest. The Kruskal-Wallis test was used for intergroup comparisons of parameters that did not show normal distribution, and the Dunn test was used for paired comparisons. One-way ANOVA test for intergroup comparisons of normally distributed parameters; the Tukey test was used for pairwise comparison of groups with equal variances, and the Tamhane test was used for pairwise comparison of groups with unequal variances. Spearman correlation test was used in correlation analyses. A p-value of <0.05 was considered statistically significant.

## Results

The general characteristics and routine laboratory parameters of the study groups are presented in Table 1.

**Table 1:** Comparison of clinical data. a: Statistically significant difference compared to the healthy control group (p<0.001)<br>b: Statistically significant difference compared to the IFG group (p<0.001)<br>c: Statistically significant difference compared to the IGT group (p<0.001)<br>d: Statistically significant difference compared to the healthy control group (p<0.05)<br>e: Statistically significant difference compared to the healthy control group (p<0.01)<br>f: Statistically significant difference compared to the IFG group: (p<0.05)<br>g: Statistically significant difference compared to the IGT group: (p<0.01)

Variables	Control Group<br>(n = 20)<br>x̄±SD	IFG Group<br>(n=20)<br>x̄±SD	IGT Group<br>(n = 20)<br>x̄±SD	DM Group<br>(n = 20)<br>x̄±SD
Age (yil)	49.5±6.1	49.1 ±5.3	49.25±7.6	51.9±3.7
Fasting glucose (mg/dL)	87.1 ±7.6	109.8±6.0^a^	109.9±8.1^a^	139.1 ±22.3^a,b,c^
Insulin (μU/mL)	7.0±2.4	10.9±5.4^d^	12.6±4.7^a^	15.1 ±8.4^e^
BMI (kg/m^2^)	24.6±2.9	27.8±3.9	30.9±4.3^a^	30.9±5.4^a^
Waist-to-hip ratio	0.87±0.08	0.88±0.09	0.89±0.09	0.91 ±0.1
Triglycerides (mg/dL)	107.2±83.9	160.3±110^e^	194.2±165.5^e^	195.3±63.4^a^
Total Cholesterol (mg/dL)	168.5±17.6	206.4±40.4^e^	213.7±35.4^a^	214.6±35.3^e^
HbA1c (%)	5.4±0.3	5.6±0.2	5.9±0.3^a,f^	6.9±0.9^a,b,g^
HOMA-IR	1.51 ±0.5	2.94±1.4^e^	3.42±1.28^a^	5.19±3.01^a,f^

FBG, insulin, triglyceride, total cholesterol, and HOMA-IR values were significantly different in all groups compared to the control group. HbA1c levels did not differ significantly between the IFG and control groups. FPG and HbA1c levels were significantly higher in the T2DM group compared to the IFG, IGT, and control groups. HbA1c levels were significantly higher in the IGT group compared to the IFG group. HOMA-IR levels were also significantly higher in the T2DM group compared to the IFG group.

The age and waist-to-hip ratio did not differ among the groups, whereas BMI levels were significantly higher in the IGT and T2DM groups than in the controls.

Table 2 shows the distribution of study variables among the groups. hsCRP levels were significantly higher in the IGT and T2DM groups compared to the control group. RBP4 and LCN2 levels did not show a difference between the groups.

**Table 2:** The distribution of study variables between the groups. *: Statistically significant difference compared to the healthy control group (p<0.01)<br>**: Statistically significant difference compared to the healthy control group (p<0.01)

Variables	Control Group<br>(n = 20)<br>x̄±SD	IFG Group<br>(n = 20)<br>x̄±SD	IGT Group<br>(n = 20)<br>x̄±SD	DM Group<br>(n = 20)<br>x̄±SD
hsCRP (mg/L)	0.09±0.17	0.41 ±0.63	0.57±0.7*	0.52±1.17**
RBP4 (ng/mL)	72.87±13.17	64.5±12.27	70.23±17.58	65.48±23.48
Lipocalin-2 (ng/mL)	3.27±0.95	3.96±1.64	3.94±1.73	3.92±1.81

The correlation analysis revealed significant correlations between hsCRP and waist-to-hip ratio (r=0.51 p<0.05), hsCRP and HbA1c (r=0.58 p<0.05), and serum RBP4 and LCN2 (r=0.65 p<0.05) in the IFG group. There was a positive correlation between serum hsCRP and lipocalin-2 levels in the T2DM group (r=0.47 p<0.05). The control group showed a positive correlation between serum RBP4 and HbA1c levels (r=0.55 p=0.01).

## Discussion

Disruptions at the receptor and post-receptor levels are the most frequently encountered factors during insulin re sistance development. Human studies investigating the relationship between various adi-pokines and insulin resistance have yielded conflicting results. These discrepancies may be attributed to differences in methodology, the recruitment of heterogeneous groups, and individual responses to inflammatory mediators and complex metabolic pathways, which are still being studied.

In this study, we analysed serum levels of RBP4, LCN2 and hsCRP in patients diagnosed with IFG, IGTand T2DM and a control group and their relationships with other variables of insulin resistance, carbohydrate metabolism and homeostasis.

Inflammation and inflammatory markers, such as CRR have been implicated in the development of insulin resistance, glucose intolerance, and T2DM. However, it remains unclear whether the relationship between T2DM and inflammatory markers is due to increased adipose tissue and obesity or is a phase in the disease pathogenesis.

Research indicates that oxidative stress from poor glycémie control leads to low-grade systemic inflammation in type 2 diabetics, with inflammatory markers decreasing when glycémie control is achieved [18]. Additionally, advanced glycosylation products, which are by-products of hyperglycemia, and even glucose itself, can increase the production of inflammatory cytokines like IL-6 and CRP [19].

In our study, we observed significantly higher levels of hsCRP in the diabetic group compared to the control group. Similarly, the IGT group also exhibited considerably elevated hsCRP levels relative to the control group, supporting the notion that CRP is a crucial factor in the pathogenesis of T2DM. Additionally, a statistically significant positive correlation was found between hsCRP and HbA1C values in the IFG group. Among the three groups in our study, IFG represents the mildest form of glucose dysrégulation, suggesting that inflammatory processes begin as soon as hyperglycemia begins.

Extensive epidemiological studies have shown that elevated levels of RBP4 are present in individuals with prediabetes and T2DM, with these levels being positively correlated with adipose tissue mass, metabolic disturbances, and cardiovascular disease [20] [21]. Additionally, a gain-of-function polymorphism in the RBP4 gene promoter region, which increases RBP4 expression in adipose tissue, is associated with an 80% increased risk of developing T2DM [22]. In insulin resistance, the expression of glucose transporter 4 (GLUT4) is down-regulated in adipocytes, leading to impaired insulin-stimulated glucose transport and worsening glucose intolerance. The genetic knockout of GLUT4 in mice adipocytes also results in increased serum RBP4 levels [23].

In our study, we aimed to determine whether serum RBP4 levels correlate with the magnitude of insulin resistance. However, no significant difference was found between the control and study groups. Interestingly, serum RBP4 levels were correlated with LCN2 levels in the IFG group. Combined with the positive correlation between hsCRP and HbA1c, this suggests that the relationships between these mediators may be independent of insulin resistance levels, with metabolic consequences emerging early prior to the progression to diabetes.

Huang et al. also reported that RBP4 could impair pancreatic β-celI function, potentially contributing to the onset and development of T2DM [24]. Studies have reported that treatment with pioglitazone and rosiglitazone significantly decreases serum RBP4 levels. Additionally, insulin-resistant obese mice can be treated with a synthetic retinoid-based RBP4 antagonist, such as fenretinide, which reduces serum RBP4 levels and improves insulin sensitivity [25]. Therefore, novel medications targeting impaired beta-cell function in individuals with IFG may help prevent the progression to IGT and, subsequently, T2DM.

Al-Absi et al. demonstrated that plasma LCN2 levels were not affected by diabetes, suggesting that observed changes in T2DM patients may be due to obesity rather than the diabetic state [26]. Furthermore, the correlation between RBP4 levels and BMI, as well as the waist-to-hip ratio, is more pronounced in individuals with obesity. The mean BMI of individuals analysed in this study is lower than in previous reports, supporting this evidence.

Mahfouz et al. [27] suggested that RBP4 could be a valuable marker for monitoring the development and progression of diabetic nephropathy. Akbay et al. [28] found that while serum RBP4 concentrations were similar in DM patients and control subjects, levels were significantly higher in T2DM patients with micro- and macroalbuminuria compared to those with normal albuminuria. This elevation may result from the filtration and reabsorption of RBP4 in the glomeruli and proximal tubules. Park et al. [29] also observed that higher circulating RBP4 levels were associated with increased urinary RBP4 levels. Additionally, Li et al. [30] reported that each 1 pg/mL increase in RBP4 was associated with a 5% increase in the risk of diabetic retinopathy (DR), suggesting that elevated RBP4 levels are linked to DR in T2DM patients. Lowering RBP4 could thus be a potential treatment strategy for these complications. However, in our study, we did not categorise T2DM patients based on the presence of diabetic complications, which might have affected our results. Comparing urinary levels of these biomarkers could also provide a better understanding of their roles in different stages of insulin resistance and T2DM.

In our study, we observed a positive correlation between serum hsCRP and LCN2 levels in the T2DM group. LCN2 exhibits a dual role in metabolic disorders, with studies suggesting different functions for the molecule during inflammation. Despite its proinflammatory nature, LCN2 can also act as an antiinflammatory agent by modulating PPARg activity and inhibiting NF-kB activity. Molecular studies reported that lipocalin-2 may contribute to the development of atherosclerosis by inducing inflammation. Additionally, clinical and experimental evidence has demonstrated the association between LCN2 levels and microvascular complications of diabetes, including diabetic neuropathy. Given the complex nature of diabetes, inflammation, atherosclerosis, and adipose tissue accumulation, along with the interplay among various adipocytokines secreted by white and brown adipose tissue, conventional single-stage measurements of these molecules may yield conflicting results. High-throughput proteomic and microarray approaches are necessary to identify and characterise the biomolecules involved in the development and progression of DM and its related complications at different stages of the condition.

Our study has several limitations that should be acknowledged and considered. Firstly, the sample size is relatively small, and we did not conduct serial measurements of circulating and urinary levels of biomarkers. Additionally, we did not categorise the T2DM group based on the presence of complications, duration of diabetes, or insulin intake. The use of exogenous insulin may have influenced insulin levels and, subsequently, HOMA-IR values. Secondly, geneleadtic variants may be associated with different circulating concentrations of these biomarkers, and we did not investigate genotype-phenotype relationships. Furthermore, commercial ELISA kits may not distinguish between exosomal and free portions of these molecules, potentially leading to conflicting results.

In conclusion, our study adds to the growing body of evidence on inflammatory markers, adipocytokines, and insulin resistance in individuals with impaired glucose metabolism and T2DM. Future research with larger sample sizes and longitudinal designs is warranted to understand further the roles of these biomarkers in the development and progression of T2DM and its complications, as well as their potential use as promising drug targets and diagnostic and prognostic markers.

## Dodatak

### Conflict of interest statement

All the authors declare that they have no conflict of interest in this work.
